# Strategies for Human Tumor Virus Discoveries: From Microscopic Observation to Digital Transcriptome Subtraction

**DOI:** 10.3389/fmicb.2016.00676

**Published:** 2016-05-13

**Authors:** Ezra D. Mirvish, Masahiro Shuda

**Affiliations:** ^1^Department of Dermatology, University of Pittsburgh Medical Center, PittsburghPA, USA; ^2^Cancer Virology Program, University of Pittsburgh Cancer Institute, University of Pittsburgh, PittsburghPA, USA

**Keywords:** tumor virus discoveries, methods, history, digital transcriptome subtraction, Merkel cell polyomavirus

## Abstract

Over 20% of human cancers worldwide are associated with infectious agents, including viruses, bacteria, and parasites. Various methods have been used to identify human tumor viruses, including electron microscopic observations of viral particles, immunologic screening, cDNA library screening, nucleic acid hybridization, consensus PCR, viral DNA array chip, and representational difference analysis. With the Human Genome Project, a large amount of genetic information from humans and other organisms has accumulated over the last decade. Utilizing the available genetic databases, Feng et al. (2007) developed digital transcriptome subtraction (DTS), an *in silico* method to sequentially subtract human sequences from tissue or cellular transcriptome, and discovered Merkel cell polyomavirus (MCV) from Merkel cell carcinoma. Here, we review the background and methods underlying the human tumor virus discoveries and explain how DTS was developed and used for the discovery of MCV.

## Introduction

Approximately 20% of human cancers worldwide are associated with infectious agents, including parasites, bacteria, and viruses (Parkin, 2006). In 12% of cancers, seven different viruses have been causally linked to human oncogenesis (**Table 1**): Epstein–Barr virus (EBV), hepatitis B virus (HBV), human T-lymphotropic retrovirus type 1 (HTLV-1), high-risk human papillomaviruses (HPV), hepatitis C virus (HCV), Kaposi’s sarcoma herpesvirus (KSHV), and Merkel cell polyomavirus (MCV). The epidemiological and clinical information provides clues that indicate whether an infectious agent is involved in the development of cancer. Cancers that are related to immunosuppression, for example, are candidates for being caused by tumor viruses (Grulich et al., 2007). During the 20th century, various methods, ranging from the classical electron microscopic observation to the advanced molecular biology techniques, were used to identify cancer-causing viruses. Here, we will review the background and methods underlying the tumor virus discoveries during the past century as well as the newest virus discovery strategy, digital transcriptome subtraction (DTS) that we used to discover MCV.

**Table 1:** Discovery methods of human tumor viruses.

Virus	Initial discovery method	Year	Associated human cancers	Reference
Epstein–Barr virus	Electron microscopic observation	1964	Burkitt’s lymphoma, Hodgkin’s lymphoma, post-transplantation lymphoproliferative disorder, and nasopharyngeal carcinoma, gastric carcinoma	Epstein et al., 1964
Hepatitis B	Immunology	1967	Hepatocellular carcinoma (HCC)	Blumberg et al., 1967
HTLV-1	Cell culture	1980	Adult T cell leukemia	Poiesz et al., 1980
Papillomavirus (high risk 16 and 18)	Nucleotide acid hybridization	1983–1984	Cervical carcinoma, head and neck carcinoma, anogenital cancer	Durst et al., 1983; Boshart et al., 1984
Hepatitis C	cDNA cloning	1989	HCC	Choo et al., 1989
Kaposi’s sarcoma associated herpesvirus	Representational difference analysis	1994	Kaposi’s sarcoma, primary effusion lymphoma, Castleman’s disease	Chang et al., 1994
Merkel cell polyomavirus	Digital transcriptome subtraction	2008	Merkel cell carcinoma	Feng et al., 2008

## Methods Used for the Human Tumor Virus Discoveries

### Cell Culture and Electron Microscopy

By the early 1960s, many viruses were known to cause tumors in animals (Javier and Butel, 2008), but none had been identified in human cancers. Burkitt (1958) first described his eponymous lymphoma in 1958 as a sarcoma involving the jaws of African children and hypothesized that the cancer’s geographic distribution might implicate some infectious agent as an etiologic factor in disease development. When the first Burkitt’s lymphoma-derived cell line was established *in vitro* and examined by electron microscopy, obvious herpesvirus morphology was readily observed (Epstein et al., 1964). Biological examinations demonstrated that this was a new human herpesvirus, which became known as the EBV after the cell line in which it was discovered (Epstein et al., 1965).

The human T-lymphotropic virus type 1 (HTLV-1), the first known human retrovirus, was also identified in cell culture (Poiesz et al., 1980). Retrovirus particles with type C morphology were observed in thin-section electron micrographs of fixed, pelleted cellular materials from two T-cell lymphoblastoid cell lines (HUT102 and CTCL-3) and peripheral blood lymphocytes from a patient with a cutaneous T-cell lymphoma. Mature particles were 100–110 nm in diameter, and consisted of an electron-dense core separated by an electron-lucent region from an outer membrane. Similar type C particles were identified in MT-1 cells derived from Japanese patients with adult T cell leukemia (ATL), which is now known to be caused by HTLV-1 (Hinuma et al., 1981). Indirect immunofluorescence staining was also used in the studies of ATL. A unique antigen was identified in MT-1 cells derived from ATL that was not found in other human lymphoid cell lines. All 44 examined ATL patients had positive serological reactions for this antigen.

In general, it is very rare that human cancer arises directly from the acute consequences of viral infection. Tumorigenesis usually occurs after a latency period of 15–40 years. However, a special exception is the X-chromosome-linked lymphoproliferative disorder (XLP) found in some EBV-infected patients. Mutated immune response genes leave susceptible males unable to respond to interferon (IFN) signaling, which can in turn lead to the development of an acute lymphoproliferative disease as a consequence of EBV replication (Kutok and Wang, 2006). Most viruses do not replicate efficiently in common laboratory cell lines due to the innate immune system, which controls IFN signaling. By targeting molecules in the IFN signaling pathway through knockout technology or RNA interference, it is possible to produce cells that are permissive for viral replication. Cell culture models with a defective IFN signaling pathway are useful for novel virus identification and characterization, especially in monitoring for cytopathic effects (CPE). The robust replication of a virus often gives rise to CPE, which involves morphological changes in the host cell, such as cell rounding, disorientation, swelling or shrinking, detachment from the culture surface, and cell death, and may indicate that the cells being studied are a good model in which to amplify the genome of the putative virus. This strategy was successfully used in acute infection with African green monkey kidney cells (Vero, a IFN-deficient cell) to identify previously uncharacterized human coronavirus (HCoV-NL63; van der Hoek et al., 2004). Humanized mice have also been developed to study viral infection in a systemic setting (Lassnig et al., 2005). In this study, viral replication was markedly enhanced in the offspring of mice with human viral receptor genes that were crossed with IFN unresponsive Stat1^-/-^ mice. Such cell and animal models, which take advantage of advanced techniques in molecular biology and genetic engineering, have the potential to provide novel insights into the biology of otherwise unculturable infectious agents and to identify the tumorigenic potential of a virus.

### Immunologic Methods to Detect Viral Antigen

Tissue culture, animal inoculation, and other virological methods were all applied unsuccessfully in early attempts to identify HBV (Blumberg, 1977). Blumberg et al. (1965) performed a systematic study of the sera collected from leukemia and hemophilia patients who had experienced frequent blood transfusions in order to detect precipitating iso-antibodies against unknown antigens found in donor serum. The ‘Australia antigen’ was identified in the serum of an Australian Aborigine as being a target of precipitating antibodies generated in the serum of transfusion patients (Blumberg et al., 1965). It was found to be rare in normal Americans, but was common in normal people from Africa, Asia, and Oceania. These data led some to hypothesize a relationship between leukemia, the unknown agent, and genetic polymorphism(s) in Australia. Subsequently, the Australian antigen was revealed to be an antigen shared by multiple hepatitis patients (Blumberg et al., 1967; Prince, 1968). Virus-like particles containing ‘Australian antigen’ on their surface were eventually found by electronic microscopy in the blood of three patients with ‘serum hepatitis’ (Dane et al., 1970; Jokelainen et al., 1970). These findings were the first indication that the disease now known as hepatitis B is caused by a virus (HBV).

The discovery of HCV was an extraordinary achievement, because isolation of the viral genome did not rely on previously visualized viral particles, growth of the virus *in vitro*, or development of an immunological assay. Patients who had transfusion-associated hepatitis were described, despite being negative for hepatitis A and B antigens, which suggested the existence of another, unidentified infectious agent (Feinstone et al., 1975). Although several immunologic and serologic assays were developed, none was a specific, reliable serologic marker for this unknown hepatitis antigen. However, administration of the putative infectious reagent induced non-A, non-B hepatitis in chimpanzees, which confirmed that an infectious agent was likely responsible for the disease (Tabor et al., 1978). A decade after the development of the chimpanzee disease model, Alter and Houghton (2000), utilizing novel methodologies in molecular biology, conducted a blind immunoscreen using a recombinant phage expression library. In searching for an unknown virus in these non-A, non-B hepatitis patients, a randomly primed cDNA library was constructed from the plasma of chimpanzees that had been injected with serum from non-A, non-B hepatitis patients. cDNA was inserted into the bacteriophage λgt11 and expressed in *Escherichia coli*. The expressed proteins were then screened against serum from an infected non-A, non-B hepatitis patient. Among millions of the clones screened, one clone encoded an antigen that showed specific seroreactivity in non-A, non-B hepatitis patients. The putative virus was shown to contain a positive-strand RNA molecule of about 10,000 nucleotides that belongs to the *Flaviviridae* family (Choo et al., 1989). Novel virus discovery by screening recombinant expression libraries from infected tissues against patient sera represented a significant breakthrough. However, library screenings are also laborious, as hundreds of millions of bacterial cDNA clones had to be screened (Alter and Houghton, 2000).

### Cross-hybridization to Identify Related Viruses

The *Papillomaviridae* comprise a super-family with several 100 members, most of which were identified in birds and mammals (de Villiers et al., 2004). Papillomaviruses are highly host-tropic and tissue-specific, causing small benign tumors known as papillomas or warts. HPV particles were observed by electron microscopy in cervical dysplasia (Della Torre et al., 1978; Meisels et al., 1981), whereas in cervical intraepithelial lesions and cervical cancer specimens, virus particles are not generally observed due to the viral integration into host genome. High-risk HPV 16 was isolated from an invasive cervical cancer by cross-hybridization with HPV type 11 DNA under non-stringent conditions (Durst et al., 1983). Using virus-specific probes, HPV 16 presence was confirmed in precursor lesion of malignant tumors (Ikenberg et al., 1983). High-risk HPV18 was subsequently identified by low stringency hybridization with HPV 8, 9, 10, and 11-related sequences (Boshart et al., 1984). zur Hausen’s (1999) research group identified both HPV16 and 18, which together are responsible for approximately 70% of invasive cervical cancers (de Sanjose et al., 2010). His group further demonstrated that HPV DNA is integrated into the host genome in cervical cancer cell lines and that viral E6 and E7 genes are expressed in cervical cancer tumors (Schwarz et al., 1985).

Nucleic acid hybridization with a single or few probes is restricted by its ability to detect only a limited number of candidate viruses. To obviate this problem, Virochip was invented to detect a broad spectrum of viruses in a single analysis (Wang et al., 2002). The Virochip was successfully used to identify a previously uncharacterized coronavirus isolated from a SARS patient during the outbreak of the severe acute respiratory syndrome (SARS; Rota et al., 2003). For the tumor virus identification, the Virochip was used to screen RNA samples from prostate tumors. A novel gammaretrovirus, Xenotropic murine leukemia virus-related virus (XMRV), which is closely related to the xenotropic murine leukemia viruses (MuLVs), was found in patients with prostate cancers (Urisman et al., 2006), and chronic fatigue syndrome (Lombardi et al., 2009). XMRV is detected in malignant prostate epithelium by using quantitative PCR assay and immunohistochemistry with an anti-XMRV specific antiserum, raising the possibility of the virus may indirectly support tumorigenesis (Schlaberg et al., 2009). However, subsequent reports disprove the etiologic connection between XMRV infection and these two diseases (Fischer et al., 2008; Hohn et al., 2009; Lee et al., 2012). XMRV was found to have arisen during the passage of a human prostate cancer in mice, as a result of recombination between two endogenous MuLVs from the mouse cells. The resulting XMRV infected human prostate cancer cells due to its xenotropic host range (Paprotka et al., 2011).

With the widespread usage of PCR and availability of extensive viral sequences, it is possible to combine short-primer hybridization and the power of PCR to amplify potential viral sequences. This method is commonly known as consensus sequence-base PCR or degenerate PCR. Primer sequences are designed based on a conserved region of a viral genome. Human hepatitis G virus was identified by amplifying a segment of a putative helicase gene with consensus primers from hepatitis A, B, and C virus (Simons et al., 1995a). As with genomic nucleotide acid hybridization, consensus PCR/degenerate PCR relies on known viral sequences, and as such has limitations in identifying novel viruses. Moreover, false-positives due to non-specific amplification and PCR contamination may introduce costly and fruitless downstream analyses. Thus, careful optimization of reaction and primer annealing temperatures and the use of nested or semi-nested strategies, as well as the use of known viruses as positive controls, are necessary.

### Differential Display Strategy in Viral Pathogen Discovery

The recent molecular techniques for virus discovery are solely based on nucleic acids—rather than conventional viral particle morphology, *in vitro* cell culture, or serological assays—but must be used in conjunction with strong epidemiologic evidence (Gao and Moore, 1996). These methods identify discrepancies in nucleotide sequence between disease-associated tissue and normal tissue by subtractive strategies. Representational difference analysis (RDA) combines physical subtractive hybridization with gene amplification to detect differences between tumor genomic DNA and that of normal cells (Lisitsyn et al., 1993). Epidemiologic evidence indicated that Kaposi’s sarcoma may be caused by a pathogen other than human immunodeficiency virus (HIV; Beral et al., 1990, 1992). By using RDA, novel viral fragments were identified in AIDS patients and further characterized as human Kaposi’s sarcoma associated herpesvirus (KSHV; Chang et al., 1994; Moore et al., 1996). RDA has also been used to successfully identify hepatitis G virus (Simons et al., 1995b) and Torque Teno virus (TTV) in non-A–E hepatitis patients (Nishizawa et al., 1997). While RDA was used to successfully identify these viruses, this technique relies heavily on the non-diseased tissue controls and is not quantitative.

### *In Silico* Subtraction Approach Using Nucleic Acid Database

With initiation of the Human Genome Project, the nucleic acid databases for humans and many other organisms rapidly expanded. Taking advantage of this new wealth of data, Weber et al. (2002) developed an *in silico* subtraction approach capable of identifying non-human sequences in ‘human’ expressed-sequence tag (EST) libraries. In this computational subtraction technique, sequences with high similarity to the human genome are subtracted from cDNA libraries, such that the remaining data are enriched for sequences of non-human origin. Weber et al. (2002) directly identified multiple human tumor viruses erroneously deposited as human ESTs in GenBank, including EBV, HBV, HPV type 16 and 18, HCV, and KSHV. Viral sequences were also found in data deposited from hepatocellular carcinoma (HCC) and cervical carcinoma cell lines: HBV sequences represented 0.1% of deposited HCC data, while HPV18 sequences accounted for 0.03% of the cervical carcinoma library. The same group also generated a cDNA library from a patient with post-transplant lymphoproliferative disorder (PTLD; Xu et al., 2003). A total of 27,840 cDNA sequences were generated and filtered by computational subtraction against known human sequences, leaving 32 non-matching sequences. Among these sequences, 10 (accounting for 0.03% of total sequences analyzed) were from EBV viruses. Taken together, these results suggest that it is possible to identify viral sequences by sampling fewer than 10,000 sequences.

## Development of Digital Transcriptome Subtraction

**FIGURE 1:**
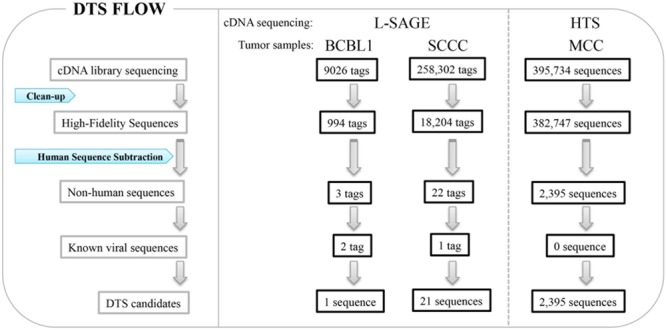
**Digital transcriptome subtraction (DTS).** L-SAGE libraries were constructed from BCBL1, a KSHV positive cell line and squamous cell conjunctival carcinoma (SCCC), and short cDNA tags were sequenced for whole transcriptome analysis. High throughput sequencing (HTS) was performed on cDNA libraries from human Merkel cell carcinoma (MCC).

Feng et al. (2007) developed DTS as a quantitative means to search for viral infection in human cancers (**Figure 1**) (Feng et al., 2007). High quality sequences are extracted with a high stringency score (Phred > = 20) to avoid sequencing errors, and all sequences artificially used in the cDNA library preparation, such as sequencing primers, are removed. All sequences matching human databases are computationally subtracted to leave ‘non-human’ candidates. There are approximately 200,000 mRNA transcripts in a cell (Bishop et al., 1974; Velculescu et al., 1999), which correspond to five transcripts per million (TPM) for a single transcript per cell. In theory, one can analyze the whole transcriptome to search for viral etiology in human cancers. Thus, if no viral transcript is found, the possibility of a virus expressing transcript in a given tumor can be excluded above a certain threshold level.

Long serial analysis of gene expression (L-SAGE) was used to sample the cellular transcriptome for the presence of viral transcripts. L-SAGE quantitatively concatenates ∼21 bp cDNA tags from the 3′ end of mRNA transcripts, allowing for the measurement of gene expression via high-throughput sequencing (**Figure 1**) (Saha et al., 2002). When they performed a pilot analysis of DTS on 9,026 SAGE tags from an expression library of BCBL-1 cells infected with KSHV, only three candidate sequences were identified as being of non-human origin: two of these sequences belonged to KSHV transcripts and the third belonged to an unannotated human expression sequence tag, as confirmed by additional experiments. Overall, 0.24% of transcripts from this cell line were of viral origin. DTS was then applied to 258,302 SAGE tags from squamous cell conjunctival carcinoma (SCCC), a cancer strongly associated with immunosuppression (**Figure 1**). Only 21 of these sequences did not align to human databases, excluding one KSHV tag as an internal control. All 21 candidates were ruled out as viral sequences by further experimental examination (Feng et al., 2007). This analysis shows that it is unlikely that distinguishable viral transcripts are present in SCCC at 20 TPM or higher, which is the equivalent of approximately four transcripts per cell.

## Merkel Cell Polyomavirus Discovery By DTS

We further performed high-throughput sequencing on cDNA libraries constructed from Merkel cell carcinoma (MCC), a malignant skin tumor frequently found among immunosuppressed, transplant, and AIDS patients (Feng et al., 2008). A total of 395,734 sequences were subtracted through DTS analysis, leaving 2,395 ‘non-human’ sequences (**Figure 1**). One transcript was similar to but distinct from African green monkey lymphotropic polyomavirus (LPV) and human BK polyomavirus T antigen sequences, defining a new polyomavirus, MCV. Subsequently, we sequenced the complete closed circular genome of MCV (5,387 bp), which encodes a T antigen locus, late gene cassette for viral capsids, and replication origin sequences. MCV is the first human member of the murine polyomavirus subgroup, and shares highest homology with LPV and a new polyomavirus recently isolated from orangutans (Groenewoud et al., 2010). Overall, DTS is a simple screening method to discover novel viral nucleic acids in a quantitative manner with high throughput sequencing. We found MCV transcripts in MCC at a level of 10 TPM or approximately five transcripts per cell and can exclude distinguishable viral transcripts in SCCC at 20 TPM or higher, which is the equivalent of approximately four transcripts per cell. This provides, for the first time, quantitative evidence against some classes of viral etiology when no viral transcripts are found, thereby reducing the uncertainty involved in new pathogen discovery.

## MCV is the First Human Polyomavirus Causing MCC

Virus discovery is only the first step to determining the etiology behind a disease. The detection of nucleic acid is not sufficient to prove causality. Our initial discovery of MCV demonstrated that the MCV genome is clonally integrated in ∼80% of MCC tumor cell DNA (Feng et al., 2008). Similar to other polyomaviruses, the MCV T antigen locus encodes for three major overlapping transcripts: large T (LT), small T (sT), and 57 kT antigens (Shuda et al., 2008). With respect to other well-studied polyomavirus T antigens, MCV LT has conserved N-terminus DnaJ and Rb binding domains, as well as C-terminus origin binding (OBD) and helicase domains, which are required for viral replication. MCV sT has a conserved PP2A binding domain (Shuda et al., 2008). Our viral sequence analysis in MCC tumors revealed that integrated MCV LT antigens are C-terminally truncated by tumor-specific mutations, which results in the loss of helicase function, whereas MCV sT remains intact (Shuda et al., 2008). This indicates that MCV is replication-defective in tumors and not a passenger virus that secondarily infects MCC tumors. We developed monoclonal antibodies, which specifically recognize MCV LT or MCV sT and carried out immunohistochemistry using these antibodies in MCC tissue microarrays. We identified that approximately ∼70% of MCC tumors express the truncated LT and sT proteins consistent with their role as directly transforming oncoproteins in MCC cancers (Shuda et al., 2009, 2011).

We identified three MCC cell lines harboring clonally integrated MCV, expressing both truncated LT and sT proteins (Houben et al., 2010; Guastafierro et al., 2013). If MCV is a direct viral carcinogen that causes most cases of MCC, we anticipate that its viral oncogene expression is necessary for the growth of MCC cells. The panT antigen knockdown (both LT and sT) by shRNA results in G0/G1 cell cycle arrest in MCV-positive MCC tumor cells and non-apoptotic cell death as measured by caspase cleavage (Houben et al., 2010). To further assess the importance of individual T antigens in MCC, we exploited an shRNA that knocks down sT antigen alone. Knockdown of MCV sT expression abolished tumor cell growth capacity (Shuda et al., 2011). On the other hand, Houben et al. (2015) demonstrated that shRNA knockdown of MCV LT alone also inhibits MCV-positive MCC cell proliferation. These results indicate that both MCV sT and LT play critical roles in the maintenance of a tumorigenic phenotype in MCC cells.

Our *in vitro* transformation studies demonstrated that MCV sT expression alone is sufficient to transform immortalized rodent fibroblast cell lines in a soft agar assay, while MCV LT expression was not (Shuda et al., 2011). MCV LT expression, however, promoted cell proliferation in human fibroblasts through its Rb binding domain (Cheng et al., 2013; Richards et al., 2015). Further, multiple groups demonstrated the oncogenic activities of MCV T antigen in transgenic mouse models. The MCC-derived MCV T antigen expression in mice, driven by the keratin 14 promoter induces papillomatosis (Spurgeon et al., 2015). MCV sT expression using a keratin 5 promoter also induces hyperproliferative lesions (Verhaegen et al., 2015). We have also demonstrated that MCV sT expression by the ubiquitin promoter in p53 null mice induces high-grade tumors in spleen and liver tissues (Shuda et al., 2015). These results are consistent with MCV being a human tumor virus encoding T antigen viral oncoproteins.

Among 13 known human polyomaviruses, eleven have been identified over the past 9 years by molecular technologies and next generation DNA sequencing (DeCaprio and Garcea, 2013; Rinaldo and Hirsch, 2013; Mishra et al., 2014). While JC virus and SV40 are suspected to play a role in human cancers, the viral copy numbers in the associated tumors are very low (less than one copy per cell; Gordon et al., 2002; Rollison et al., 2005). Recently, to survey for the presence of human polyomaviruses in cancer, Toptan et al. (2016) developed the immunohistochemistry-based pan-human polyomavirus screening that can detect T antigen protein expression of all known human polyomaviruses including SV40. However, the study did not find any evidence of the polyomavirus T antigen expression in the 1,184 various tumor cases except the MCV T antigen expression in MCC tumors (Toptan et al., 2016). Thus MCV is the only polyomavirus strongly linked to a human cancer at present.

## Concluding Remarks

Identification of ‘non-human’ sequences from a whole transcriptome does not necessarily indicate the presence of other genomes in human disease, as contamination from sample preparation may introduce some ‘non-human’ sequences. The existence of such sequences is only meaningful in the context of a strong association with human disease. Koch’s postulates and their revisions provide a valid standard for judging disease causation (Fredricks and Relman, 1996). In spite of acute infection, tumorigenesis usually occurs after a long latency period. To move from association to causation for putatively carcinogenic viral agents, zur Hausen (1999) has also proposed new criteria for defining a causal role of an infection in human cancers: (1) strong epidemiological plausibility and evidence that a viral infection represents a risk factor for the development of a specific tumor; (2) the consistent presence and persistence of the ‘non-human’ sequences in cells of the tumor; (3) the stimulation of cell proliferation by the ‘non-human’ genome (or part of it) in corresponding cell culture systems; (4) the demonstration that the ‘non-human’ genome induces proliferation and the malignant phenotype of the tumor. To address whether MCV is causally relevant to MCC, additional evidence is needed to elucidate tumorigenic potential of the MCV T antigen.

In addition to the cancers known to be caused by human viruses, there are many tumors in which a viral etiology is suspected, including some hematopoietic malignancies, breast cancer, colorectal cancer, basal cell carcinoma of the skin, and lung cancers in non-smokers (Zur Hausen, 2009). Undoubtedly, DTS analysis on whole transcriptomes of human tumors, in conjunction with other approaches of the sort described above, will help us to identify new clues or eliminate potential viral etiologies. Despite the power of DTS in searching for viral etiologies, there are some caveats that should be kept in mind: (1) DTS relies on high database quality and unique features of non-human sequences. Viral sequences that have been accidentally deposited into human databases or viral sequences that are indistinguishable from human transcripts will cause analyses to fail. A specific subgroup of human endogenous retrovirus (HERV-K) is suspected to be associated with multiple cancers (Moyes et al., 2007). DTS against the human genome would subtract out endogenous retroviral sequences as human and thus fail to detect pathogenic sequences. (2) Viral transcripts that lack polyadenylation sites, such as found in *Flaviviridae* and *Reoviridae*, cannot be detected. (3) Viruses causing tumors without gene expression, such by insertional mutagenesis, will be missed. Thus, extra efforts should be made to overcome the caveats of DTS analysis by combining with other methods for virus discovery.

The invention of high throughput sequencing technology developed massive transcriptome database generated from various human cancers in the Cancer Genome Atlas (TCGA), which could be a direct resource for DTS. Several studies interrogated transcriptome sequencing data from nearly 4,000 different tumors generated within the TCGA consortium for the presence of viral sequences by using the strategy similar to DTS (Khoury et al., 2013; Tang et al., 2013). One of these studies revealed a rare bladder cancer with BK polyomavirus integration that expresses full length LT antigen (Tang et al., 2013) and also identified a novel enterovirus in colon adenocarcinoma which is unlikely to be oncogenic. The use of DTS to the expanding transcriptome database in the TCGA may allow us to discover the eighth human tumor virus in the near future.

## Author Contributions

All authors listed, have made substantial, direct and intellectual contribution to the work, and approved it for publication.

## Conflict of Interest Statement

The authors declare that the research was conducted in the absence of any commercial or financial relationships that could be construed as a potential conflict of interest.
